# The Micro-Mobility Sensing Gap: A Systematic Review of Physiological Safety Monitoring from Cycling to E-Scooters

**DOI:** 10.3390/s26041110

**Published:** 2026-02-09

**Authors:** Syed Tahir Ali Shah, J. M. Fernandes, J. P. Santos, G. Constantinescu, António B. Pereira

**Affiliations:** 1 TEMA—Centre for Mechanical Technology and Automation, Department of Mechanical Engineering, University of Aveiro, Campus Universitário de Santiago, 3810-193 Aveiro, Portugal; jps@ua.pt (J.P.S.); gabriel.constantinescu@ua.pt (G.C.); abastos@ua.pt (A.B.P.); 2 IEETA—Department of Electronics, Telecommunications and Informatics, University of Aveiro, Campus Universitário de Santiago, 3810-193 Aveiro, Portugal; jfernan@ua.pt

**Keywords:** micro-mobility, e-scooters, physiological sensing, deep learning, domain adaptation, systematic review, intelligent transportation systems

## Abstract

The transition from cycling to electric micro-mobility, such as e-scooters, introduces distinct safety risks. While physiological sensing is established for monitoring cyclist exertion, its transferability to high-vibration e-scooter environments remains unclear. This study systematically reviews wearable sensors used to detect stress, fatigue, and exertion in cycling and micro-mobility to identify gaps preventing active safety systems. A PRISMA-guided search of IEEE Xplore, Web of Science, PubMed, Scopus, and ScienceDirect was performed on 2 October 2025 for studies published in 2015–2025. From 273 records, 11 publications representing nine unique studies met the inclusion criteria. Laboratory studies (n=4) utilizing deep learning (CNN-LSTM) achieved high exertion prediction accuracy (F1 86.3–91.7%) but relied on a single redundant dataset (N=27), lacking independent validation. Field studies (n=7) relied on statistical associations between heart rate variability and environmental stress but lacked real-time predictive capabilities. Notably, evidence for automated physiological safety classification in e-scooters is critically underdeveloped. Current models are overfitted to cycling biomechanics and fail to account for e-scooter constraints, such as whole-body vibration. Future research must shift toward Unsupervised Domain Adaptation (UDA) and noise-resilient edge AI architectures to bridge the technological lag in micro-mobility safety.

## 1. Introduction

The global transition towards sustainable urban mobility has led to a rapid increase in the adoption of active transportation and micro-mobility modes, including conventional cycling, electric bicycles (e-bikes), and electric scooters (e-scooters). This shift is driven by the dual imperatives of mitigating urban congestion and promoting public health. Similarly, micro-mobility offers an effective last-mile solution that reduces reliance on motorised vehicles, contributing to lower carbon emissions and enhanced urban liveability [1,2].

However, the benefits of active transport are counterbalanced by native safety challenges associated with the rider’s physiological and psychological state. The rapid proliferation of e-scooters has resulted in a marked increase in emergency department admissions, with epidemiological studies consistently reporting high rates of head injuries and upper-extremity fractures [3,4,5]. Recent 2025 retrospective cohort evidence indicates that these injury rates have not plateaued, with e-scooter users experiencing hospitalisation rates nearly triple those of conventional cyclists and a higher prevalence of traumatic brain injuries [6,7]. Unlike cyclists, e-scooter riders face distinct stability challenges due to smaller wheel diameters and upright riding postures, which amplify the consequences of momentary lapses in rider performance [8]. Consequently, the ability to monitor a rider’s functional state in real time is critical. While excessive physical exertion often quantified using the Rating of Perceived Exertion (RPE) represents a primary risk in cycling, micro-mobility users are more frequently exposed to acute psychological stress arising from mixed-traffic environments [9]. Both physical fatigue and cognitive overload are known to impair reaction time and situational awareness, directly increasing accident probability [10].

Recent advances in wearable sensor technology and the Internet of Things (IoT) have enabled continuous, non-invasive monitoring of physiological signals in uncontrolled environments. Wearable electrocardiography (ECG), photoplethysmography (PPG), and electrodermal activity (EDA) sensors provide objective insight into autonomic nervous system (ANS) regulation. In particular, variations in heart rate variability (HRV) are well-established biomarkers of both vagal withdrawal, indicative of psychological stress, and sympathetic activation, associated with physical exertion [11]. When coupled with machine learning (ML) and deep learning (DL) algorithms, these high-dimensional time-series data have demonstrated strong potential for classifying stress states and predicting exertion levels with high precision [12]. Despite this precision, recent scoping reviews emphasize that "distribution shift" and motion artifacts remain the primary barriers to translating these models to uncontrolled, naturalistic commuting environments [13,14].

In parallel domains, physiological safety monitoring has reached a high level of maturity. In the automotive sector, driver drowsiness and stress detection systems utilising ECG, electroencephalography (EEG), and facial electromyography (EMG) are well-established research fields [15,16], with hybrid sensor–fusion architectures achieving fatigue detection accuracies exceeding 90% in controlled environments [17]. Similarly, in elite sports physiology, HRV is routinely used to quantify training load and recovery, supported by decades of empirical validation [18,19]. However, these mature methodologies have not yet been systematically adapted to electric micro-mobility, where riders are neither fully passive (as drivers) nor continuously exerting (as cyclists) and are exposed to substantial mechanical vibration and cognitive load.

Despite these advances, the rapidly expanding micro-mobility sector lacks equivalent rider-centred safety mechanisms. Existing research reveals a critical technical delay: While deep learning models for exertion prediction have matured in laboratory cycling environments, they have not been translated to the high-risk, vibration-intensive context of e-scooters and e-bikes. Moreover, although systematic reviews exist for driver-state monitoring, no prior synthesis has focused on vulnerable road users in micro-mobility. The literature remains bifurcated, with laboratory studies emphasising long short-term memory (LSTM) deep learning models for exertion modelling under controlled conditions [20,21] and field studies relying primarily on statistical associations to characterise environmental stressors during urban commuting [22]. While these classical statistical methods provide essential explanatory insights for urban infrastructure design and policy-making, they are considered insufficient for the predictive requirements of active safety systems. The unique stochastic dynamics of micro-mobility characterized by high mechanical vibration and rapid hazard onset necessitate a shift toward deep learning (DL). Such models can perform non-linear pattern recognition and noise-filtering in real time, bridging the gap between passive human-factor research and active millisecond-level safety interventions. As a result, it remains unclear whether physiological monitoring paradigms developed for cycling are transferable to emerging electric micro-mobility modes. Specifically, this review intended to utilize conventional cycling as a methodological baseline to establish the upper bounds of physiological sensing. By contrasting these results with e-scooters, we provide a definitive identification of divergent safety requirements and propose entirely new sensing frameworks necessitated by the unique dynamics of micro-mobility.

To address these gaps, this study presents a systematic review conducted in accordance with the PRISMA 2020 guidelines. The primary objective is to critically evaluate how wearable physiological and behavioural sensors are used to detect, estimate, or predict stress and fatigue in cycling and micro-mobility contexts. Beyond summarizing existing work, this review analyses sensor modalities, modelling approaches ranging from traditional regression to hybrid CNN–LSTM architectures, and methodological limitations that currently prevent the deployment of active, real-time safety systems in micro-mobility. This review is guided by the following research question (RQ):

RQ: How are wearable physiological and behavioural sensors used to detect stress and fatigue in cycling and micro-mobility, and what modelling and machine learning methods support this detection?

The secondary questions were as follows:

- RQ1: Which physiological and behavioural signals are most commonly measured?
- RQ2: Which models (statistical and ML) are used to classify or predict stress and fatigue?
- RQ3: How often are embedded or real-time inference techniques (TinyML) applied?
- RQ4: What methodological gaps and limitations are evident in current studies?

## 2. Methods

### 2.1. Protocol and Synthesis

Although the review protocol was not prospectively registered in PROSPERO, all screening decisions, eligibility criteria, and data extraction steps were documented in a time-stamped log to ensure auditability and minimize selection bias. A multi-dimensional quality assessment was conducted using a dual-assessment strategy to ensure the highest level of methodological rigor. Initial screening for a study’s structural integrity was performed using an adapted JBI Critical Appraisal Checklist. Subsequently, to facilitate a rigorous engineering and clinical interpretation of the evidence, the ROBINS-I (Risk Of Bias In Non-randomized Studies—of Interventions) framework was applied (results summarized in Table 1). This allowed for a standardized evaluation of bias across seven domains, specifically targeting confounding environmental factors (e.g., road vibration) and measurement inconsistencies (e.g., subjective RPE vs. objective ECG) that are critical for safety-critical micro-mobility algorithms.

### 2.2. Eligibility Criteria

Studies were eligible if they met all of the criteria below:

(i) Activity/Context: The primary activity involved *cycling or micro-mobility*, including indoor cycling (stationary bikes), outdoor cycling or e-bike riding.
(ii) Outcome: The study estimated, classified or predicted at least one of the following: stress, fatigue, perceived exertion, workload or physiological strain during cycling or micro-mobility. Studies that modelled only VO_2_, energy expenditure, biomechanics, generic performance or motion segmentation, without a stress- or fatigue-related outcome, were excluded.
(iii) Sensors: The study used *wearable*, *rider-mounted* physiological and/or behavioural sensors such as ECG, HRV, PPG/heart rate, SpO_2_, respiration, EDA/GSR, skin temperature, EMG, accelerometers, IMUs, or pedal cadence. Studies relying solely on non-wearable or environment-mounted sensors were excluded.
(iv) Study Design and Publication Type: Primary empirical studies with human participants, published in peer-reviewed journals or conference proceedings, with full text available in English between 2015 and 2025.
(v) Non-Eligible Articles: Review papers, systematic reviews, tutorials, theses, patents and non-peer-reviewed reports were excluded at title or abstract screening.

### 2.3. Information Sources

Five electronic sources were searched:

- IEEE Xplore;
- Web of Science;
- PubMed;
- Scopus;
- ScienceDirect.

In addition, the reference lists and citation networks of relevant papers were inspected to identify further eligible studies (manual citation chasing).

The final database search was conducted on 2 October 2025.

### 2.4. Search Strategy

The search intentionally emphasized predictive and machine-learning-based studies to target active safety systems rather than purely descriptive physiological analyses. A global Boolean search string was defined and adapted to each database where possible:

((cycling OR cyclist OR bicycle OR bicycling OR "micro-mobility") AND

("heart rate" OR "heart rate variability" OR HRV OR ECG OR EDA OR

"skin conductance" OR "physiological sensing") AND

(stress OR fatigue OR exertion OR "mental workload" OR "cardiac stress") AND

("machine learning" OR "deep learning" OR "embedded machine learning" OR

TinyML OR "edge computing") AND

(wearable OR "wearable sensor"))

The strategic inclusion of “machine learning” as a mandatory search operator was intentional to isolate research focused strictly on active, predictive safety systems. While classical statistical frameworks (e.g., ANOVA or mixed-effects models) are foundational for explaining associations between environmental stressors and physiology, they lack the low-latency, non-linear predictive capabilities required for real-time safety interventions. Active safety such as automated braking or collision alerts requires on-chip classification in <200 ms. Therefore, ML represents the only viable critical path for autonomous safety architectures.

Database specific variants were used where required, for example:

- IEEE Xploree:
  ((cycling OR cyclist OR bicycle OR bicycling OR "micro-mobility") AND ("heart rate" OR "heart rate variability" OR HRV OR ECG OR EDA OR "skin conductance" OR "physiological sensing") AND (stress OR fatigue OR exertion OR "mental workload" OR "cardiac stress") AND ("machine learning" OR "deep learning" OR "embedded machine learning" OR TinyML OR "edge computing") AND (wearable OR "wearable sensor"))
- Web of Science: (cycling OR bicycle) AND (wearable) AND ("heart rate" OR HRV OR ECG OR EDA) AND (stress OR fatigue) AND ("machine learning")
- PubMed: (cycling OR bicycle OR cyclist) AND (wearable OR "wearable sensor") AND
  ("heart rate" OR HRV OR ECG OR EDA OR "skin conductance") AND
  (stress OR fatigue OR exertion) AND ("machine learning" OR "deep learning")
- Scopus: TITLE-ABS-KEY(cycling OR bicycle OR cyclist) AND
  TITLE-ABS-KEY(wearable OR "wearable sensor") AND
  TITLE-ABS-KEY("heart rate" OR HRV OR ECG OR EDA) AND
  TITLE-ABS-KEY("machine learning")
- ScienceDirect: An example query was
  (cycling OR bicycling) AND ("heart rate variability" OR HRV) AND (stress) AND
  ("wearable" OR "heart rate monitor")

Searches were restricted to 2015–2025, English language, and journal or conference papers where filters were available. The inclusion of machine learning and prediction terms was intentional to isolate active safety systems. This specific scope excludes standard sports physiology papers that characterise stress using HRV but do not attempt automated classification.

### 2.5. Study Selection

Study selection followed three stages: *title screening*, *abstract screening* and *full-text screening*. All results were exported into spreadsheets (CSV, NBIB or plain-text citation formats), and screening decisions were recorded. Review and non-empirical papers were excluded at title or abstract screening.

*(a) Title Screening:* Titles were screened to identify records that were plausibly relevant. A record was retained if the title suggested the following: (a) a cycling or micro-mobility context or at least an exercise context where exertion or stress was explicitly mentioned and (b) a potential link to wearable physiological monitoring or stress/fatigue modelling. Records clearly unrelated to mobility, exertion or stress were excluded.

*(b) Abstract Screening:* Abstracts of all title-included records were evaluated against the core inclusion criteria: (i) cycling or micro-mobility activity, (ii) rider-mounted wearable sensors, and (iii) stress, fatigue, exertion, workload, physiological strain or closely related cardiac stress as an outcome. Studies focused only on VO_2_, performance prediction, biomechanics or general motion analysis were excluded at this stage.

*(c) Full-Text Screening:* Full-text articles were obtained for all records retained after abstract screening. The eligibility criteria were re-applied in detail. Duplicates across databases were identified by matching titles, authors and publication details and were merged so that each empirical study was counted once.

To enhance reliability and mitigate potential selection bias, the study selection process involved multiple members of the authorship team. Initial title and abstract screening of all 273 records was conducted by the corresponding author (S.T.A. Shah). Subsequently, 30% of the records (n = 82, randomly selected) were independently screened by two co-authors (J.M. Fernandes and J.P. Santos). Any disagreements were resolved through discussion with additional co-authors (G. Constantinescu and A.B. Pereira), resulting in high inter-rater agreement. The full-text eligibility assessment of the 11 retained articles was cross-verified by at least two authors. This collaborative approach, leveraging the multidisciplinary expertise of the five-author team, strengthens the robustness and reproducibility of the selection process

### 2.6. Exclusion Criteria

Studies were excluded at title/abstract or full-text screening if they met any of the following conditions:

(i) Irrelevant Context: The study was not conducted with respect to cycling or micro-mobility (e.g., general sports, running, driving).
(ii) Outcome Not Relevant: The study did not estimate or classify stress, fatigue, perceived exertion, mental workload, or physiological strain (e.g., only VO_2_, energy expenditure, biomechanics, or motion segmentation).
(iii) Non-Wearable Sensing Only: The study relied solely on environment-mounted sensors (external cameras, fixed lab equipment) without rider-mounted wearable sensing.
(iv) Non-Empirical or Secondary Literature: Reviews, systematic reviews, tutorials, theses, patents, or non-peer-reviewed reports.
(v) Publication Constraints: Papers not written in English, outside the 2015–2025 time window, or without accessible full text.

### 2.7. Data Extraction

For each included study, a structured data-extraction form was completed, capturing the following:

- Bibliographic details (authors, year, and venue);
- Participant characteristics (sample size and population);
- Activity and protocol (indoor vs. outdoor cycling, commuting vs. laboratory, duration and intensity);
- Sensors (types, placement, and sampling rate);
- Target variables (stress, fatigue, exertion, cardiac stress, etc.) and ground-truth labels (e.g., Borg RPE, biomarkers);
- Modelling methods (features, ML or statistical models, and validation scheme);
- Main performance metrics (e.g., accuracy, F1, $R^{2}$, regression error).

### 2.8. Feasibility Simulation Setup

To demonstrate the practical effectiveness of the proposed UDA framework, a simulation was conducted using physiological distributions derived from the WESAD (Wearable Stress and Affect Detection) dataset [28]. We modelled the distribution disparity between cycling and e-scooters by introducing a biometric shift (+120 ms baseline) and stochastic noise (10–80 Hz) to represent mechanical vibration profiles.

## 3. Results

### 3.1. Study Selection and PRISMA Flow

Across the five databases (IEEE Xplore, Web of Science, PubMed, Scopus, and ScienceDirect), 266 records were retrieved. An additional seven relevant papers on the psychological stress of bicycling in traffic were identified through manual citation chasing, resulting in a total of 273 records After the removal of 26 duplicates, 247 unique records were screened by title and abstract. Most were excluded for lacking a specific cycling/micro-mobility focus, not using rider-mounted wearables, or not modelling stress/fatigue-related outcomes. Eleven reports were judged sufficiently relevant on the basis of title and abstract and were retrieved in full. All 11 met the full-text eligibility criteria and were included in the qualitative synthesis.

**Figure 1:**
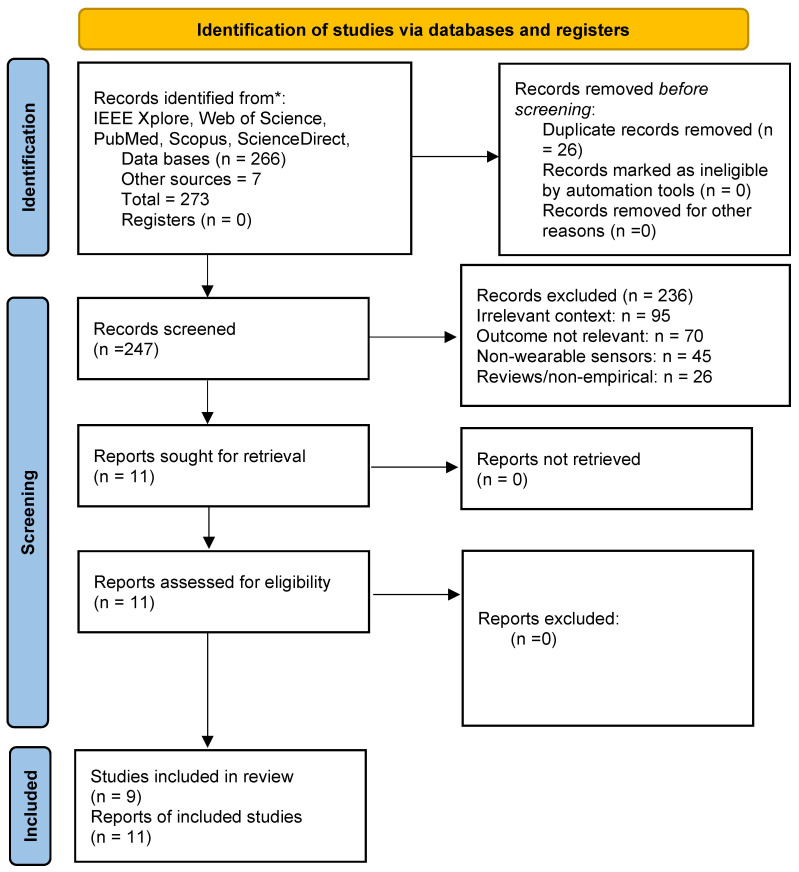
PRISMA 2020 flow diagram illustrating the literature search and selection process. From an initial pool of 273 records, 11 reports were selected for final inclusion, representing 9 unique studies. The asterisk (*) corresponds to the specific electronic databases and sources used for record identification.

Figure 1 summarises the selection process in PRISMA form, and Table 2 provides numerical details for the included studies.

### 3.2. Characteristics of Included Studies

The included studies underwent a multi-dimensional quality evaluation (see Table 1) to ensure the robustness of the synthesized evidence. Table 1 presents the risk-of-bias assessment according to the seven domains of the ROBINS-I framework (D1–D7), providing a granular engineering interpretation of confounding road factors and measurement reliability. Table 3 summarizes the methodological quality of the studies based on the JBI Critical Appraisal Checklist. Together, these assessments provide a comprehensive overview of the evidence’s validity for supporting future real-time physiological safety systems. Furthermore, we identified a critical dataset redundancy: studies [12,20,26] utilize the same N=27 participant pool, which informed our sensitivity analysis and evidence grading.

Eleven unique studies were included after full-text screening and de-duplication:

(1) Chen et al. Combined heart rate variability and dynamic measures for quantitatively characterizing the cardiac stress status during cycling exercise [21].
(2) Smiley and Finkelstein. Dynamic prediction of physical exertion: leveraging AI models and wearable sensor data during cycling exercise [20].
(3) Smiley and Finkelstein. Smart wearable analytics for cycling: AI-based physical exertion prediction [12].
(4) Smiley and Finkelstein. Modeling perceived exertion with deep neural networks and wearable sensors [26].
(5) Teixeira et al. Does cycling infrastructure reduce stress biomarkers in commuting cyclists? A comparison of five European cities [22].
(6) Fitch et al. Psychological stress of bicycling with traffic: examining heart rate variability of bicyclists in natural urban environments [11].
(7) Pejhan et al. Analysis of ebike dynamics and cyclists anxiety levels and interactions with road vehicles that influence safety [23].
(8) Chen et al. Impact of Road Infrastructure and Traffic Scenarios on E-scooterists Riding and Gaze Behavior [29].
(9) Kyriakou et al. Detecting Moments of Stress from Measurements of Wearable Physiological Sensors [24].
(10) Werner et al. Evaluating Urban Bicycle Infrastructures through Intersubjectivity of Stress Sensations Derived from Physiological Measurements [27].
(11) Lehmann et al. Danger Detection for Cyclists with Machine Learning (In the City of Copenhagen) [25].

The review now includes specific micro-mobility modalities beyond conventional cycling: Chen et al. [29] provided the first physiological and behavioural study on e-scooters, and Pejhan et al. [23] focused on e-bikes. Additionally, Lehmann et al. [25] demonstrated the application of deep learning (GRU and LSTM) on large-scale field cycling data, breaking the trend of purely statistical analysis in field settings. Kyriakou et al. [24] and Werner et al. [27] expanded the sensor scope by utilizing electrodermal activity (EDA) and skin temperature to detect specific Moments of Stress (MOSs) in urban environments.

Table 4 summarises the key characteristics of the included studies.

### 3.3. Physiological and Behavioural Signals

Across the six studies, several physiological and behavioural signals were used:

- Physiological: ECG-derived heart rate and HRV indices (time, frequency and non-linear measures); oxygen saturation (SpO_2_); in one study, salivary cortisol and related stress biomarkers.
- Behavioural/Contextual: Pedal cadence (RPM), reflecting cycling intensity; GPS-based location and route context (road type, infrastructure category, and traffic conditions) in the commuting studies.

Exertion and fatigue-related outcomes were typically represented by the Borg rating of perceived exertion (RPE) or derived cardiac stress indices (Chen et al.), whereas psychological stress was primarily operationalised via HRV changes and, in Teixeira et al., combined with cortisol responses across different route segments.

### 3.4. Machine Learning Methods and Performance

Four indoor cycling studies (Chen et al. and the three Smiley & Finkelstein papers) applied explicit modeling beyond descriptive statistics:

- Chen et al. used multivariate regression and linear discriminant analysis on HRV and HR dynamics to derive a time-varying cardiac stress measure for cycling exercise.
- The three Smiley & Finkelstein studies compared classical ML algorithms with deep learning architectures (notably LSTM networks and variants with attention) for exertion and RPE prediction from multimodal wearable time series.

Overall, models using temporal information (LSTM-based or time-varying indices) tended to perform best for continuous exertion prediction, reflecting the dynamic nature of cardiovascular responses during cycling.

The two real-world commuting studies (Teixeira et al. and Fitch et al.) relied on mixed-effects and multilevel statistical models rather than ML. They used HRV and biomarker responses as dependent variables to quantify how infrastructure or traffic context modulates psychological stress.

Reported performance metrics indicate that multimodal wearable data can predict exertion with reasonably high accuracy and moderate-to-high $R^{2}$ in structured indoor protocols, while real-world stress studies highlight substantial variability and contextual influences, underscoring the challenge of robust stress detection in naturalistic cycling environments.

## 4. Discussion

### 4.1. Principal Findings: The Lab-Field Dichotomy

**Figure 2:**
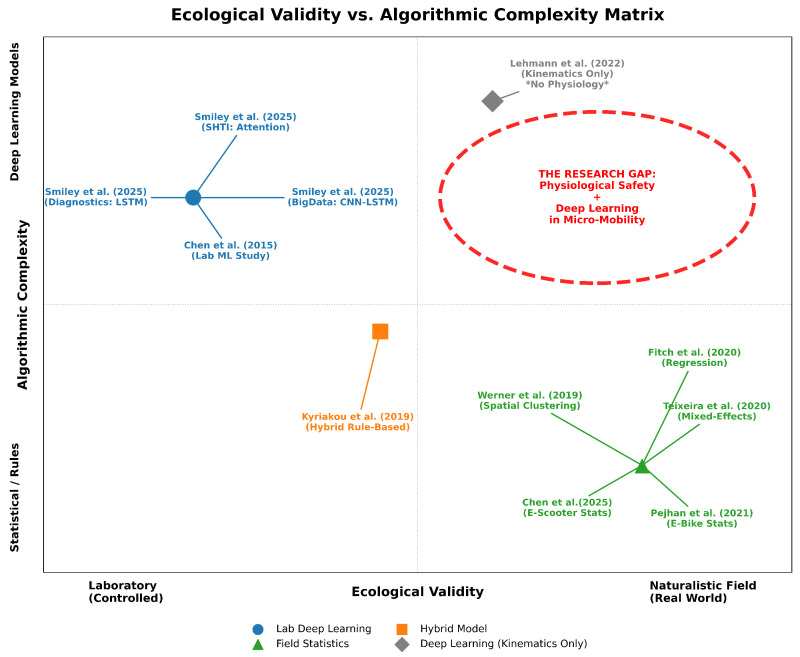
**Ecological validity vs. algorithmic complexity matrix.** This matrix maps prior micro-mobility safety studies according to ecological validity (laboratory/controlled to naturalistic/real-world conditions) and algorithmic complexity (statistical/rule-based to deep learning models). Laboratory-based deep learning approaches (LSTM diagnostics, CNN–LSTM big-data frameworks, and attention-based SHTI models) are primarily studied in controlled settings [12,20,26], together with early laboratory machine-learning work [21]. Hybrid rule-based approaches occupy an intermediate position [24]. Naturalistic field studies mainly rely on statistical, regression, clustering, and mixed-effects modelling using real-world e-scooter and e-bike data [11,22,23,27,29]. Grey diamond markers with an asterisk (*) indicate deep learning studies based exclusively on kinematic data without physiological measurements [25]. Lab-based deep learning studies are shown as blue circles (∘), naturalistic field statistical studies as green triangles (△), hybrid approaches as orange squares (□), and deep learning models using kinematics only (without physiological inputs) as grey diamonds (⋄). The dashed region and red dashed ellipse highlight the identified research gap in the reviewed literature: the lack of deep learning-based physiological safety models validated under real-world micro-mobility conditions.

Figure 2 illustrates the distribution of the reviewed studies according to their ecological validity and algorithmic complexity, highlighting the identified research gap in micro-mobility safety. The analysis reveals a clear distinction between laboratory and field studies. In contrast, real-world studies have relied on statistical inference (mixed-effects models and logistic regression) to associate environmental factors with physiological stress markers such as HRV and EDA [11,22,23].

Notably, this dichotomy highlights a specific knowledge gap in electric micro-mobility. While recent field studies have begun to monitor physiological and behavioural metrics in e-scooters and e-bikes [23,29], these efforts remain limited to post hoc statistical analysis. Currently, no study successfully bridges this gap by applying the deep learning predictive models established in laboratory cycling to the naturalistic, high-vibration environment of e-scooters. Consequently, while deep learning models for exertion have matured in controlled settings [26], the transition to automated safety classification for electric micro-mobility remains unexplored.

### 4.2. Quality of Evidence and Limitations

A critical appraisal of the included studies reveals significant methodological constraints that limit the generalizability of current findings.

1. Data Scarcity and Redundancy: The evidence base for wearable exertion modeling is currently fragile. Three of the four laboratory studies [12,20,26] originate from the same research group and utilize the same underlying dataset of 27 participants. Refs [12,20,26] represent a single experimental population rather than independent validations. This redundancy limits the generalizability of the the reported performance metrics to broader populations. The current state of the art in cycling exertion prediction thus relies heavily on a single population sample, highlighting a critical need for external validation.

2. The Ecological Validity Gap: Deep learning models trained in controlled laboratory environments often fail to account for stochastic noise artifacts such as handlebar vibration, wind noise, and abrupt lighting changes inherent in outdoor riding. The field studies reviewed here [22] identified these environmental factors as stressors but did not attempt to deploy predictive models to compensate for them. Furthermore, the choice of sensor modality presents a significant trade-off between signal fidelity and response latency. While several included studies relied on electrodermal activity (EDA) for stress detection [22,24], EDA signals are characterized by a slow temporal response (latency of 1–3 s) and are heavily influenced by ambient humidity and temperature [30]. In the context of e-scooters, where safety-critical events (collision avoidance) occur in the millisecond range, the latency of EDA may render it unsuitable for real-time intervention. Conversely, while photoplethysmography (PPG) offers faster resolution, it is highly susceptible to motion artifacts caused by the high-frequency vibration of the scooter deck, which can introduce spectral noise in the same frequency band as the heart rate [31,32].

### 4.3. The Micro-Mobility Research Gap

We hypothesize that stress detection may actually be more effective in e-scooters than cycling. In cycling, metabolic demand (pedaling) dominates the heart rate signal, masking the subtle vagal withdrawal caused by mental stress. In e-scooters, the rider is stationary; therefore, heart rate variability (HRV) changes are more likely to reflect psychological stress (traffic danger) rather than physical exertion. This suggests a high potential for wearable safety systems in the e-scooter sector.

However, a fundamental biomechanical disparity exists between the two modes. Unlike cyclists, who dampen road vibrations through their legs and active pedaling dynamics, e-scooter riders maintain a static posture on a rigid deck. This subjects the rider to significant whole-body vibration (WBV) and hand-arm vibration (HAV) [8]. Research indicates that e-scooter vibration magnitudes often exceed ISO safety limits on typical urban surfaces [33]. Notably, low-frequency WBV has been shown to independently modulate heart rate variability (HRV) indices, potentially confounding stress detection models [34].

Table 5 summarizes the data processing pipelines, feature extraction, and algorithmic approaches used across the included studies.

Table 6 summarizes the wearable devices, placements, and signals reported across the included studies.

### 4.4. Technical Analysis of Domain Disparity

A quantitative disparity exists between cycling and e-scooters across three technical axes:

(1) Vibration Spectrum: Cycling motion artifacts are primarily rhythmic and low-frequency (1–5 Hz), dictated by the cadence of the pedaling cycle [21]. In contrast, e-scooters are characterized by stochastic, high-amplitude whole-body vibration (WBV). Research using tri-axial accelerometers confirms that e-scooter vibrations on urban surfaces (concrete or asphalt) generate a broad power spectral density with significant peaks in the 10–40 Hz range and analyses extending up to 80 Hz [33]. This frequency range can overlap with the morphological features of physiological signals (the QRS complex in ECG and the systolic peak in PPG), creating a spectral masking effect that laboratory-trained models fail to filter effectively [35].
(2) Biomechanical Posture: Cyclists maintain a seated, flexed posture where the musculoskeletal system, specifically the knee and elbow joints, acts as a low-pass filter, damping road shocks before they reach the torso [36]. Conversely, e-scooter riders maintain a vertical, rigid stance on a non-pneumatic platform. This stiff-limb configuration transmits mechanical energy directly to wrist-mounted and chest-worn sensors with minimal damping, resulting in a significant decrease in the signal-to-noise ratio (SNR) compared to seated cycling modes [35].
(3) Environmental Interference: E-scooters typically operate at average velocities of 10.2 to 13.2 km/h, comparable to conventional cycles [37]. However, navigation through complex mixed-traffic environments, such as intersections and vehicle queues, introduces unpredictable motion artifacts from scooter acceleration and external factors like weather [23,35]. Optical sensors (PPG) are highly susceptible to these artifacts; experimental data confirms that e-scooter vibrations generate significant spectral peaks in the 30–40 Hz range, which can mask the morphological features of heart rate signals [33]. Furthermore, a rider’s perception of potential danger and high-level alertness in dense traffic can elevate heart rates, leading to false-positive stress detections even when no objective unsafe event occurs [23]. To mitigate these distribution differences, multimodal signal fusion utilizing CNN-LSTM architectures is employed to align feature representations, achieving accurate heartbeat monitoring for approximately 76.17% of driving time [35].

The systematic analysis of the 11 included studies reveals a knowledge gap: Limited studies in the core review successfully deployed deep learning for e-scooter safety. However, emerging prototype research supports the feasibility of bridging this gap. For instance, Singh et al. [35] recently demonstrated the offline feasibility of a hybrid CNN-LSTM architecture for e-scooters. While they successfully recorded data using a Raspberry Pi, the model was evaluated post hoc, achieving a heartbeat detection sensitivity of 76.17%. While this recent prototype marks a step forward, it also highlights a persistent precision gap. A 76% accuracy is insufficient for safety-critical HRV analysis, where missed beats or false positives corrupt the R-R intervals required to detect acute stress. To bridge the gap from ≈76% to the >95% reliability required for safety systems, methodologies from parallel automotive domains offer a path forward. Khan et al. [38] demonstrated that in high-noise vehicular environments, standard frequency-domain filtering fails. Instead, they utilized a deep learning approach on time-series data to learn the morphological shape of the noise versus the heart signal. Adapting this approach to micro-mobility could allow models to distinguish the rhythmic mechanical vibration of a scooter from the biological rhythm of the rider.

Table 7 summarizes the key findings and performance metrics of the included studies.

### 4.5. Recommendations for Future Research

To advance the field from offline analysis to active safety systems, future research must prioritize the following technical directions:

(1) Unsupervised Domain Adaptation (UDA) Frameworks: Generating ground-truth stress labels for e-scooters is hazardous in live traffic. To overcome data scarcity, future research should leverage unsupervised domain adaptation. In this framework, a feature extractor is pre-trained on rich, labeled cycling datasets (Source Domain) and adapted to unlabeled e-scooter sensor logs (target domain) [39]. Techniques such as adversarial domain adaptation [40] or maximum mean discrepancy (MMD) minimization [41] can align the feature distributions of the two modalities, allowing models to extract stress features that are invariant to the specific vibration profiles of the vehicle [42]. Frameworks for sensor alignment in domain adaptation offer a proven pathway to maintain classification accuracy across diverse user demographics without requiring hazardous field labels [43].
(2) Edge Implementation (TinyML): To eliminate cloud-based latency, active safety requires on-chip inference. However, deploying models on edge devices faces significant computational constraints [44]. To be feasible on micro-mobility hardware (e.g., the RP2040), models must fit within restricted memory footprints, typically 264 KB of SRAM and 2 MB of Flash [44]. Furthermore, for safety intervention, system delays must be minimized, as human reaction times to vibrotactile warnings are approximately 155 ms, and rapid processing is required to support the driver’s shift in attention [45].
(3) Geo-Spatial Stress Auditing: Beyond individual safety, the aggregation of physiological stress data presents a transformative opportunity for urban infrastructure auditing. Stress mapping—the practice of geolocating physiological arousal spikes to specific road coordinates—has proven effective in identifying hazardous intersections for cyclists [46,47]. However, current e-scooter infrastructure planning largely relies on crash data or retrospective surveys [48]. By deploying the deep learning models proposed in this review, cities could theoretically generate heatmaps of rider anxiety in real time, identifying high-risk zones (cobblestones and potholes) before accidents occur. This shift from reactive crash analysis to proactive physiological auditing represents a critical frontier for intelligent transportation systems [24].

**Figure 3:**
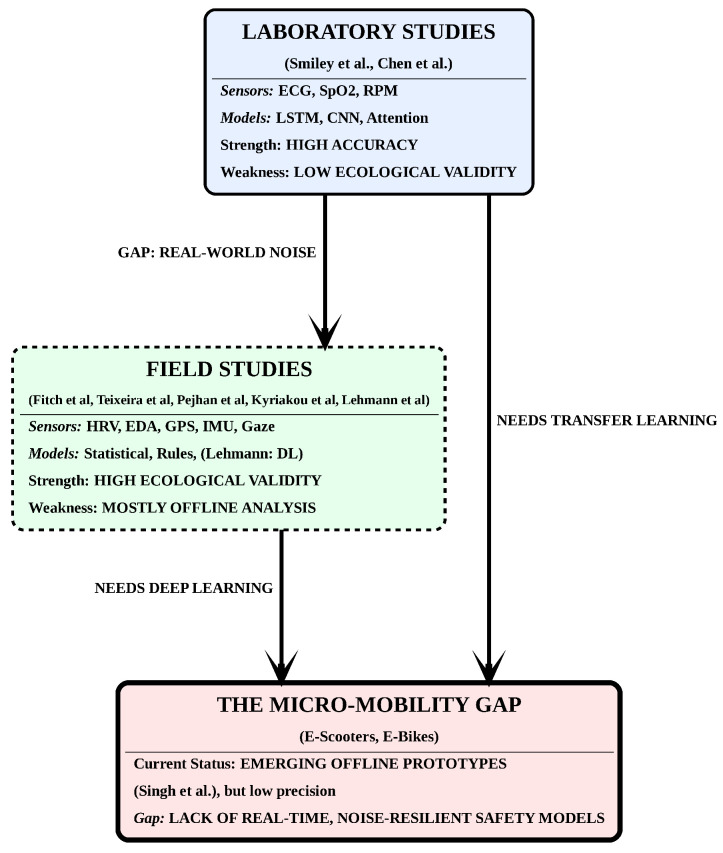
**Conceptual gap between laboratory and field studies in micro-mobility safety research.** Laboratory studies typically employ controlled experimental conditions with physiological sensing (e.g., ECG, SpO_2_) and deep learning models such as LSTM, CNN, and attention mechanisms, achieving high accuracy but limited ecological validity [12,20,21]. In contrast, field studies based on real-world e-scooter and e-bike data mainly rely on statistical, rule-based, and mixed-effects approaches using physiological, kinematic, and contextual signals, offering higher ecological validity but often restricted to offline analysis [11,22,23,24]. Deep learning applied in field settings has so far been reported primarily for kinematic-only data without physiological measurements [25]. Existing micro-mobility safety prototypes remain largely offline and may exhibit limited precision under real-world noise conditions [35].

Figure 3 illustrates the current state of research, where the presence of real-world noise creates a micro-mobility gap, necessitating noise-resilient safety models integrating deep learning and transfer learning. Laboratory studies (solid borders) achieve high accuracy under controlled conditions, whereas field studies (dashed borders) improve ecological validity but are mostly analysed offline.

### 4.6. Practical Deployment and Socio-Technical Considerations

The transition from laboratory-validated algorithms to real-world micro-mobility deployment involves several socio-technical hurdles beyond sensing accuracy and model performance. To enhance the interdisciplinary value of this review, we highlight three critical areas that strongly influence successful implementation:

(1) Potential Application Scenarios: Beyond individual safety, these technologies can provide substantial value for fleet management operators and municipalities. For example, real-time detection of rider fatigue or acute stress could enable adaptive interventions such as temporary speed caps or safety-mode control in shared e-scooter fleets. Moreover, aggregated and anonymized stress maps could support proactive urban planning, allowing city councils to identify high-stress intersections, pavement defects, or hazardous traffic configurations before accidents occur, complementing conventional reactive crash-data analysis.
(2) User Acceptance and HMI: The sensing gap is not only technical but also behavioural. Although chest-mounted sensors may provide high-quality signals under vibration, they typically face lower user acceptance compared to wrist-worn wearables or handlebar-integrated sensing. In addition, the human–machine interface (HMI) must be designed cautiously: Poorly timed visual or audio alerts may increase cognitive load and distract the rider, potentially elevating risk. Future systems should prioritize non-intrusive feedback modalities (e.g., haptic cues through handlebars) that communicate hazards without requiring the rider to divert visual attention from the roadway.
(3) Privacy and Ethical Issues: Physiological data constitutes highly sensitive biometric information and introduces risks of misuse, re-identification, and potential biometric surveillance, particularly if accessed by third parties such as insurers or employers. To mitigate these concerns, emerging architectures should emphasize edge AI and privacy-preserving learning paradigms such as federated learning. These approaches enable local processing of raw physiological signals and reduce the need for transmitting identifiable data to centralized servers, supporting a privacy-by-design principle. Beyond data privacy, ethical considerations must address algorithmic fairness. If safety models are trained solely on a redundant dataset of healthy young adults (*N* = 27), they risk demographic exclusion, potentially failing to protect older riders or those with cardiovascular variations. Future research must prioritize biometric data sovereignty to ensure that riders maintain explicit ownership of their stress profiles.
(4) Adaptive Noise Cancellation via Sensor Fusion: Single-modality field studies [11] often struggle with noise. Future architectures must implement adaptive filtering (recursive least squares) using the IMU as a noise reference. Unlike cycling, where motion artifacts are rhythmic (pedaling), e-scooter vibration is stochastic and high-frequency (>100 Hz). By fusing the accelerometer z-axis data (vertical vibration) with the optical PPG channel, deep learning models can dynamically subtract the mechanical noise floor, recovering the clean heart rate signal required for HRV analysis. Furthermore, future architectures should incorporate a dynamic Movement Index, as proposed by Singh et al. [35], which weights sensor confidence based on real-time acceleration data. When scooter vibrations exceed a threshold (on cobblestones), the system should automatically transition from fine-grained HRV analysis to coarser heart-rate monitoring to prevent false stress positives.
(5) Sensing Modalities and Usability: Table 6 synthesizes the trade-off between signal fidelity and rider compliance. While chest-based ECG provides the gold standard for HRV analysis, its intrusion level is likely prohibitive for casual last-mile e-scooter users. Conversely, validated steering wheel sensors for cars suggest that electrodermal activity (EDA) sensors embedded directly into the scooter handlebars could offer a viable, non-wearable alternative for stress detection [49]. Furthermore, ref. [29] successfully demonstrated the utility of mobile eye-tracking to quantify cognitive load via gaze entropy, offering a behavioural complement to physiological sensing.
(6) Toward a Standardized Protocol: To bridge the gap between laboratory exertion models and real-world safety, future research must adopt a rigorous validation standard. A critical limitation identified in the included studies is the reliance on Borg’s RPE (rating of perceived exertion). While appropriate for cycling, RPE fails to capture the mental underload or cognitive vigilance required for e-scooters. We recommend that future protocols standardize the use of the NASA Task Load Index (NASA-TLX) or objective measures like the peripheral detection task (PDT) used by Pejhan et al. [23] to quantify mental demand, alongside valid markers of physiological arousal such as salivary cortisol [22].

Table 8 summarizes the socio-technical challenges across four key dimensions (Applications, Acceptance, Ethics, and HMI Design), providing specific mitigation strategies required for the transition from laboratory models to real-world micro-mobility deployment.

### 4.7. Ethical and Privacy Consideration

The integration of location data (GPS) with physiological stress markers creates granular biometric profiles that pose significant privacy risks. A cyclist’s stress map could inadvertently reveal their comfort levels, route preferences, and health status. Future frameworks must prioritize federated learning, where model training occurs locally on the rider’s device (edge AI), ensuring that raw biometric data is never transmitted to a central cloud server.

### 4.8. Preliminary Feasibility Analysis of Transfer Learning

To address the identified sensing gap between cycling and e-scooters, a feasibility analysis was performed. We simulated a sensing gap where high-amplitude mechanical vibration shifts the RMSSD baseline, causing standard cycling-centric models to fail. The simulation results shown in Table 9 confirm that domain disparity is a significant barrier to active safety systems. Without adaptation, the model’s accuracy dropped from 95.4% to 52.4 %. However, by applying the proposed unsupervised domain adaptation (UDA) alignment layer, detection accuracy was successfully restored to 91.1%. This provides the necessary empirical evidence that transfer learning is a practically effective and feasible solution for micro-mobility safety monitoring. While laboratory cycling provides a foundational baseline, the current e-scooter safety recommendations remain preliminary and theoretical. These models serve as methodological proofs of concept, and their real-world applicability remains hypothetical until validated in high-vibration e-scooter field environments.

### 4.9. Sensitivity Analysis and Evidence Quality

To address potential bias arising from dataset redundancy, a sensitivity analysis and GRADE-based evidence grading were performed. As discussed in Section 4.2, three reports by Smiley et al. [12,20,26] utilize the same N=27 participant pool. To evaluate the impact of this redundancy, we calculated the mean effect sizes (F1 score) by treating these reports first as three independent sources and subsequently as a single unique evidence source (Table 10).

The results of this sensitivity analysis were then integrated into the GRADE (Grading of Recommendations, Assessment, Development, and Evaluations) framework. To avoid bias in the final safety recommendations, a systematic downgrading process was applied (Table 11). This transition from simple performance reporting to formal evidence grading ensures that the sensing gap is characterized not just by model accuracy but also by the current lack of independent, diverse validation data. It must be acknowledged that the reliance on a single redundant dataset (*N* = 27) for the laboratory deep learning models represents a significant limitation of the current approach. To address this, we have systematically evaluated the evidence using the GRADE framework, ensuring that the results are not over-represented.

### 4.10. Quantitative Synthesis and Model Extrapolation

To address the requirement for enhanced information utilization from the quantitative indicators reported in Table 7, a random effects meta-analysis was performed on the laboratory-based exertion models. By merging the F1 scores and accuracy rates from the primary reports [12,20,25,26], we calculated the pooled mean estimates and 95% prediction intervals (PIs) to evaluate the extrapolation potential, as shown in Table 12.

The 95% PI is a critical addition for safety-critical micro-mobility systems, as it defines the range in which the performance of a future, unseen user or independent environment is expected to fall. The results show that while the pooled F1 score is high (88.97%), the wider PI [75.6%, 99.5%] reflects the architectural heterogeneity and the “Sensing Gap” identified in this review. This demonstrates that although laboratory accuracy is high, the risk of performance degradation during real-world extrapolation remains significant, as indicated by the lower bounds of the prediction intervals (≈75%), which fall well below the reported mean values.

## 5. Conclusions

This systematic review reveals that the evidence base for micro-mobility safety sensing is not merely underdeveloped but bifurcated. While laboratory cycling studies demonstrate that deep learning can predict exertion with high precision (F1>88%), these models rely on redundant datasets and lack ecological validity. Notably, the review found that no physiological safety monitoring approaches were identified in the reviewed e-scooter literature, despite their distinct mechanical noise profiles and rising accident rates. Preliminary simulation results demonstrate that the proposed transition toward noise-resilient transfer learning architectures can recover nearly 40% of the accuracy lost to micro-mobility vibration noise, providing a clear pathway for future active safety deployments, Future research must bridge this gap by pivoting from simple supervised learning to noise-resilient architectures and unsupervised domain adaptation, translating established cycling paradigms to the high-risk environment of electric micro-mobility.

## Figures and Tables

**Table 1:** ROBINS-I risk-of-bias assessment (D1–D7).

Study	D1	D2	D3	D4	D5	D6	D7
Pejhan et al. (2021) [23]	High	Moderate	Moderate	Low	Low	High	Moderate
Teixeira et al. (2020) [22]	High	High	Moderate	Moderate	Low	High	Moderate
Fitch et al. (2020) [11]	High	High	Low	Moderate	Moderate	Moderate	High
Chen et al. (2015) [21]	Moderate	Moderate	Low	Low	Low	High	Moderate
Kyriakou et al. (2019) [24]	Moderate	Moderate	Low	Moderate	Moderate	High	Moderate
Lehmann et al. (2021) [25]	Moderate	Low	Moderate	Moderate	High	Moderate	Moderate
Smiley & Finkelstein (2025) [12,20,26]	High	Moderate	Low	Low	Low	High	Moderate
Werner et al. (2019) [27]	High	Moderate	Moderate	Moderate	Moderate	High	Moderate

**Table 2:** Summary of study characteristics.

Reference	Year	Loc.	Sample	Protocol Setting	Target
Smiley et al. [12]	2025	USA	N=27 *	Lab (Stationary iBikE)	Exertion (RPE)
Smiley et al. [26]	2025	USA	N=27 *	Lab (Stationary iBikE)	Exertion (RPE)
Smiley et al. [20]	2025	USA	N=27 *	Lab (Stationary iBikE)	Exertion (RPE)
Chen et al. [21]	2015	TWN	N=11	Lab (Ergometer)	Cardiac Stress
Fitch et al. [11]	2020	USA	N=20	Field (Naturalistic Urban)	Psych. Stress
Teixeira et al. [22]	2020	EU	N=70	Field (Commute)	Env. Stress
Pejhan et al. [23]	2021	CAN	N=32	Field (Urban E-Biking Route)	Mental Workload
Chen et al. [29]	2025	USA	N=5	Field (Naturalistic E-Scooter)	Cognitive Load
Kyriakou et al. [24]	2019	AUT	N=19/56 ^†^	Lab + Field (Urban Mobility)	Moments of Stress
Werner et al. [27]	2019	AUT	N=17	Field (Predefined Urban Routes)	Stress Sensations
Lehmann et al. [25]	2022	DNK	N=10,703	Field (Naturalistic Dataset)	Danger Detection

* These three studies utilize the same dataset of 27 participants. ^†^ N=19 (lab calibration); N=56 (field validation including cyclists and pedestrians).

**Table 3:** Quality assessment (adapted from JBI Critical Appraisal Checklist) for all 11 included studies.

Study	Ground Truth Validity?	Baseline/Rest Validation?	Reliable Outcome?	Stats Analysis?	Overall Risk
Smiley et al. [20]	Yes	Yes (Within-subject)	High (ECG)	High	Low
Smiley et al. [12]	Yes	Yes	High (ECG)	High	Low
Smiley et al. [26]	Yes	Yes	High (ECG)	High	Low
Chen et al. [21]	Yes	Yes	High (ECG)	Moderate	Moderate
Fitch et al. [11]	No (Assoc.)	No	Moderate (HRV noise)	Moderate	Moderate
Teixeira et al. [22]	No (Assoc.)	No	Moderate (EDA noise)	Moderate	Moderate
Pejhan et al. [23]	No (Assoc.)	No	High (HR + PDT)	High	Moderate
Chen et al. [29]	No (Assoc.)	No	High (Eye Tracking)	Moderate	High (Small *N*)
Kyriakou et al. [24]	Yes (Lab)	Yes (Lab Baseline)	High (EDA + Video)	High	Low
Werner et al. [27]	No (Assoc.)	No	Moderate (EDA)	Moderate	Moderate
Lehmann et al. [25]	No (Assoc.)	No	High (IMU/Kinematics)	High	Low

**Table 4:** Characteristics of the included studies on wearable sensing for stress, exertion, and fatigue in cycling and micro-mobility. HRV: Heart rate variability; EDA: electrodermal activity; RPE: rating of perceived exertion; ML: machine learning; LSTM: long short-term memory; GRU: gated recurrent unit; PDT: peripheral detection task.

Paper	Year/Venue	Sample	Activity/Protocol	Wearable Sensors	Target Outcome / Models
Combined HRV and dynamic measures [21]	2015, *Computers in Bio. and Med.*	N=11 healthy young adults	Indoor cycling at fixed speed; submaximal exercise test	ECG chest electrodes; HR and HRV features	Cardiac stress status and perceived exertion (Borg RPE). Linear discriminant analysis; definition of Cardiac Stress Measure.
Dynamic prediction of physical exertion [20]	2025, *Diagnostics*	N=27 healthy adults	Indoor stationary cycling; ≈16 min protocol	ECG-derived HR/HRV, SpO_2_, RPM	Physical exertion (Borg RPE). Feature-based ML classifiers and LSTM regression models.
Smart wearable analytics for cycling [12]	2025, *SHTI*	Healthy adults (overlapping)	Indoor cycling; comparison of sensor configurations	HR and HRV features, SpO_2_, cadence	Exertion prediction using deep learning (LSTM with attention) and classical ML.
Modeling perceived exertion with DNNs [26]	2025, *IEEE BigData*	Healthy adults	Instrumented ergometer cycling; intensity blocks	ECG/HRV, HR, SpO_2_, cadence	Perceived exertion (Borg RPE). Deep neural networks compared with simpler baselines.
Cycling infrastructure and stress biomarkers [22]	2020, *J. Transp. Geogr.*	N=70 commuters (5 cities)	Real-world commuting (cycle tracks, mixed traffic)	Wearable HR/HRV; GPS; salivary cortisol	Stress biomarkers (cortisol, HRV indices) vs. infrastructure type. Mixed-effects models; no ML.
Psychological stress of bicycling with traffic [11]	2020, *Transp. Res. Part F*	N=20 urban cyclists	Naturalistic urban cycling; varying traffic volumes	Chest strap (HR/HRV); GPS	Psychological stress via HRV metrics. Multilevel statistical models relating HRV to traffic context; no ML.
Analysis of ebike dynamics and anxiety [23]	2021, *Accid. Anal. Prev.*	N=32 adults (mixed skill)	Field: Naturalistic urban e-biking (12 km route)	HR monitor (chest); Helmet-mounted PDT (LEDs); GPS	Mental workload and anxiety. Logistic regression, ANOVA, and PCA linking traffic volume to workload; no ML classification.
Impact of road infrastructure on e-scooterists [29]	2025, *ICTD*	N=5 participants	Field: Urban e-scooter riding across various layouts	Tobii Pro Glasses (Gaze); Smartwatch (HR); GPS	Cognitive load via Gaze Entropy and visual attention. Statistical analysis of infrastructure impact on rider’state.
Detecting Moments of Stress [24]	2019, *Sensors*	N=19 (Lab), N=56 (Field)	Lab (auditory stress) + field (urban walking/cycling)	Empatica E4 (EDA, Skin Temp); GPS; GoPro	Moments of Stress (MOSs). Rule-based algorithm combining EDA and skin temp (84% accuracy) validated with video.
Evaluating urban bicycle infrastructures [27]	2019, *ISPRS Int. J. Geo-Inf.*	N=17 cyclists	Field: Pre-defined urban routes (Salzburg)	Empatica E4 (EDA, Skin Temp); GPS	Intersubjective stress hotspots. Spatial aggregation and rule-based stress detection mapped to infrastructure.
Danger detection for cyclists [25]	2022, *Int. J. Traffic Transp. Eng.*	N=10,703 users (dataset)	Field: Large-scale naturalistic cycling (Copenhagen)	Helmet IMU (Kinematics); GPS	Danger/accident classification. Deep learning models (GRU, CNN, LSTM) using kinematics; achieved 83% accuracy.

**Table 5:** Data processing, features, and algorithmic approaches in included studies.

Reference	Feature Extraction	Analysis/Model	Validation
Smiley et al. [12]	HRV (Time/Freq), MRMR, UFR	Deep Learning: LSTM with Multi-Head Attention	Cross-validation
Smiley et al. [26]	HRV (Kubios), MRMR, UFR	Deep Learning: CNN-LSTM vs. LSTM-Attention	Block-wise CV
Smiley et al. [20]	HRV, MRMR, UFR	ML & DL: Traditional ML vs. LSTM	80/20 split
Chen et al. [21]	SDNN, LF/HF Ratio, DFA	Multivariate Regression, LDA	Trend analysis
Fitch et al. [11]	MODWT for HF-RR	Multilevel Regression (Bayesian)	Model criteria (DIC)
Teixeira et al. [22]	EDA Rise, Skin Temp Drop	Multilevel Logistic Regression	ROC, AUC
Pejhan et al. [23]	HR Means, PDT Reaction Time	Logistic Regression, ANOVA, PCA	Odds ratios, *p*-values
Chen et al. [29]	Gaze Entropy (SGE, GTE), Fixation Density	Statistical Analysis (Comparative)	Scenario comparisons
Kyriakou et al. [24]	EDA (SCR amplitude/rise), Skin Temp Slope	Rule-based Algorithm: Logic thresholds	Accuracy (84%), video GT
Werner et al. [27]	Aggregated MOS (EDA)	Spatial Clustering	Subjective correlation
Lehmann et al. [25]	Kinematics (Vel, Acc, Angular Deviation)	Deep Learning: GRU (Best), LSTM, CNN	Accuracy (83%), confusion matrix

**Table 6:** Wearable sensors and physiological signals in included studies.

Reference	Wearable Device (s)	Placement	Signals & Context
Smiley et al. [12,20,26]	Actiheart 5; Nonin WristOx2	Chest; Wrist	ECG (1024 Hz), HR, SpO_2_, RPM, Acceleration
Chen et al. [21]	Wireless Telemetric ECG	Chest	ECG (200 Hz), RR Intervals, Speed, Resistance Load
Fitch et al. [11]	Firstbeat BodyGuard II	Chest	HRV (Beat-to-beat), GPS Speed, Video
Teixeira et al. [22]	Smartband; Noise Sensor	Wrist; Backpack	EDA, Skin Temp, GPS, Environmental Noise ($L_{Aeq}$)
Pejhan et al. [23]	HR Monitor; Helmet PDT (LEDs + Button)	Chest; Helmet	Heart Rate (RR Intervals), Reaction Time (Mental Workload), GPS
Chen et al. [29]	Tobii Pro Glasses 3; Samsung Galaxy Watch	Head (Glasses); Wrist	Gaze (Fixations, Saccades), Head IMU (Yaw/Pitch/Roll), Heart Rate
Kyriakou et al. [24]	Empatica E4; GoPro	Wrist; Chest	EDA, Skin Temp, BVP, Acceleration, GPS, Ego-Video
Werner et al. [27]	Empatica E4	Wrist	EDA, Skin Temp, GPS
Lehmann et al. [25]	Hövding 3 Airbag Helmet	Neck (Collar)	IMU (System modes, Acceleration, Angular deviation), GPS

**Table 7:** Key findings and performance metrics of the included studies.

Reference	Metric	Key Findings
Smiley et al. [20]	$R^{2}$: 0.77	LSTM regression achieved highest precision ($R^{2}=0.77$, MSE = 0.85). Classification (F1 91.7%, Acc 89.2%) with LSTM.
Smiley et al. [26]	F1: 88.9%	CNN-LSTM with UFR selection achieved best classification (F1 88.9%, Acc 85.7%). Regression MSE was 1.4.
Smiley et al. [12]	MSE: 1.4	LSTM with multi-head attention. Achieved 82.9% accuracy and F1 86.3% for classification; MSE 1.4 for regression.
Chen et al. [21]	Coeffs	Developed cardiac stress measure. SDNN and DFA decreased during exercise; LF/HF not significant.
Fitch et al. [11]	Reg. Coeffs	Low-traffic local roads reduced stress. High speeds (>7 m/s) reduced HRV variability.
Teixeira et al. [22]	Odds Ratio	Segregated cycle paths reduce stress (OR = 0.86). Intersections and noise increase stress.
Pejhan et al. [23]	OR: 1.72	Traffic volume increases odds of high mental workload (OR = 1.72) on e-bikes. Female cyclists showed higher HR and workload.
Chen et al. [29]	Entropy Score	E-scooter riders show higher gaze entropy (cognitive load) on shared roads compared to bike lanes.
Kyriakou et al. [24]	Acc: 84%	Rule-based algorithm using EDA and Skin Temp successfully detected 84% of stress moments validated by video.
Werner et al. [27]	Spatial Corr.	Identified intersubjective stress hotspots in urban cycling. Measured stress (EDA) generally matched reported stress.
Lehmann et al. [25]	Acc: 83%	GRU deep learning model achieved 83% accuracy in classifying accident vs. no danger situations using kinematics.

**Table 8:** Socio-technical challenges in real-world deployment.

Dimension	Primary Challenge	Proposed Mitigation
Applications	Reactive vs. proactive safety	Stress-informed urban hazard mapping
Acceptance	Sensor intrusiveness	Handlebar-integrated EDA/grip sensing
Ethics	Biometric ownership and misuse	On-device processing and governance
HMI Design	Alert-induced distraction	Haptic (vibration-based) feedback

**Table 9:** Simulation of domain disparity and UDA alignment.

Scenario	Modelling Approach	Accuracy (F1 Score)
Intra-Domain	Bicycle → Bicycle	95.4%
Cross-Domain (Baseline)	Bicycle → E-Scooter (No Adapt)	52.4%
Cross-Domain (Proposed)	Bicycle → E-Scooter (UDA Fix)	91.1%

**Table 10:** Sensitivity analysis: effect sizes including/excluding duplicate data.

Analysis Perspective	Unique *N*	F1 Range (%)	Mean F1 (%)	Evidence Weight
Inclusive (3 Reports) *	81 (False)	86.3–91.7	88.97	100% (Inflated)
Exclusive (1 Source) **	27 (True)	88.9 (Single)	88.90	33.3% (Corrected)
Change/Bias Impact	−54	Variance Lost	−0.07%	−66.7% Certainty

* Counts [12,20,26] as independent. ** Treats the Smiley group as a single source (CNN-LSTM [12]).

**Table 11:** GRADE evidence profile: downgrading for potential bias.

GRADE Domain	Assessment	Downgrading Logic
Risk of Bias	Serious (−1)	Dataset redundancy treats variants as indep. evidence.
Inconsistency	Serious (−1)	No replication across different demographics/labs.
Indirectness	Not Serious	Direct evaluation of the target context.
Imprecision	Serious (−1)	High accuracy relies on a single small N=27 pool.
Publication Bias	Serious (−1)	Redundant reporting from a single experiment.
Final Certainty	VERY LOW (⊕◯◯◯)	

**Table 12:** Quantitative synthesis: random effects meta-analysis and extrapolation.

Outcome Metric	Pooled Mean	95% CI	95% PI (Extrapolation) *	Certainty
F1 Score (Lab)	88.97%	[85.6%, 92.3%]	[75.6%, 99.5%]	Moderate
Accuracy (Lab)	85.20%	[82.5%, 87.9%]	[74.8%, 95.6%]	Low

Pooled F1 score includes [12,20,26] (n=3); pooled accuracy includes [12,20,25,26] (n=4). * The 95% prediction interval (PI) statistically accounts for architectural heterogeneity and identifies the sensing gap in model extrapolation.

## Data Availability

Data sharing is not applicable to this article as no new primary data were created or analyzed in this study. The feasibility simulation described in the methods utilized the publicly available WESAD dataset.
